# Supplementary material for: Single Cell Transcriptomic and Chromatin Profiles Suggest Layer Vb Is the Only Layer With Shared Excitatory Cell Types in the Medial and Lateral Entorhinal Cortex
Source: Front Neural Circuits. 2022 Jan 26;15:806154. doi: 10.3389/fncir.2021.806154 (PMC8826650; doi:10.3389/fncir.2021.806154)
Supplement: Supplementary file 14 [file Data_Sheet_1.ZIP › 2/knownResults.html]

2 - Homer Known Motif Enrichment Results


# Homer Known Motif Enrichment Results (2)

Homer *de novo* Motif Results  
Gene Ontology Enrichment Results  
Known Motif Enrichment Results (txt file)  
Total Target Sequences = 1000, Total Background Sequences = 48311

|  |  |  |  |  |  |  |  |  |  |  |  |
| --- | --- | --- | --- | --- | --- | --- | --- | --- | --- | --- | --- |
| Rank | Motif | Name | P-value | log P-pvalue | q-value (Benjamini) | # Target Sequences with Motif | % of Targets Sequences with Motif | # Background Sequences with Motif | % of Background Sequences with Motif | Motif File | SVG |
| 1 | T C G A T G A C G T A C C G T A C A G T T G A C A C G T A C T G A G C T A G C T | NeuroG2(bHLH)/Fibroblast-NeuroG2-ChIP-Seq(GSE75910)/Homer | 1e-63 | -1.453e+02 | 0.0000 | 453.0 | 45.30% | 10319.3 | 21.36% | motif file (matrix) | svg |
| 2 | T C A G A C G T T C G A T A G C A G T C C G T A A C T G G T A C A C G T A C T G A T C G A G T C | Atoh1(bHLH)/Cerebellum-Atoh1-ChIP-Seq(GSE22111)/Homer | 1e-61 | -1.422e+02 | 0.0000 | 333.0 | 33.30% | 6203.2 | 12.84% | motif file (matrix) | svg |
| 3 | C T A G T C G A T G A C A G T C C G T A A C T G G T A C A C G T A C T G A C T G | BHLHA15(bHLH)/NIH3T3-BHLHB8.HA-ChIP-Seq(GSE119782)/Homer | 1e-58 | -1.338e+02 | 0.0000 | 400.0 | 40.00% | 8743.2 | 18.10% | motif file (matrix) | svg |
| 4 | T C A G T G A C G T A C C G T A A C G T T G A C A C G T T C A G A G C T G A C T | NeuroD1(bHLH)/Islet-NeuroD1-ChIP-Seq(GSE30298)/Homer | 1e-51 | -1.197e+02 | 0.0000 | 267.0 | 26.70% | 4711.7 | 9.75% | motif file (matrix) | svg |
| 5 | T A C G T G C A A G T C C G T A A C G T T G A C A C G T A C T G A C T G G C A T | TCF4(bHLH)/SHSY5Y-TCF4-ChIP-Seq(GSE96915)/Homer | 1e-51 | -1.175e+02 | 0.0000 | 423.0 | 42.30% | 10168.2 | 21.05% | motif file (matrix) | svg |
| 6 | C T G A T G A C T G A C C G T A A C G T T G A C A G C T C T A G A C G T G A C T | Olig2(bHLH)/Neuron-Olig2-ChIP-Seq(GSE30882)/Homer | 1e-49 | -1.141e+02 | 0.0000 | 522.0 | 52.20% | 14306.8 | 29.61% | motif file (matrix) | svg |
| 7 | T G A C A G T C C G T A A C T G G T A C A C G T A C T G A C G T G A C T G A T C | Twist2(bHLH)/Myoblast-Twist2.Ty1-ChIP-Seq(GSE127998)/Homer | 1e-48 | -1.113e+02 | 0.0000 | 454.0 | 45.40% | 11626.8 | 24.07% | motif file (matrix) | svg |
| 8 | A G C T A C G T A C T G A T G C A G T C C G T A C T G A T A C G | NF1-halfsite(CTF)/LNCaP-NF1-ChIP-Seq(Unpublished)/Homer | 1e-37 | -8.631e+01 | 0.0000 | 373.0 | 37.30% | 9508.5 | 19.68% | motif file (matrix) | svg |
| 9 | C T A G T C G A A C G T A C T G C G T A A T G C A C G T G T A C C G T A A G C T G A T C G T A C | Atf3(bZIP)/GBM-ATF3-ChIP-Seq(GSE33912)/Homer | 1e-26 | -6.117e+01 | 0.0000 | 208.0 | 20.80% | 4559.6 | 9.44% | motif file (matrix) | svg |
| 10 | C T G A T C A G G T A C G C T A A C T G T G A C G C A T C A T G | SCL(bHLH)/HPC7-Scl-ChIP-Seq(GSE13511)/Homer | 1e-26 | -6.036e+01 | 0.0000 | 706.0 | 70.60% | 26102.0 | 54.03% | motif file (matrix) | svg |
| 11 | T C G A A C G T C A T G G C T A T A G C C G A T G T A C G C T A A C G T A T G C | AP-1(bZIP)/ThioMac-PU.1-ChIP-Seq(GSE21512)/Homer | 1e-25 | -5.771e+01 | 0.0000 | 220.0 | 22.00% | 5100.3 | 10.56% | motif file (matrix) | svg |
| 12 | C A G T T G C A A C G T A C T G C G T A A T C G C G A T T G A C C G T A A C G T | BATF(bZIP)/Th17-BATF-ChIP-Seq(GSE39756)/Homer | 1e-24 | -5.679e+01 | 0.0000 | 207.0 | 20.70% | 4688.0 | 9.70% | motif file (matrix) | svg |
| 13 | A C T G C T A G T C G A C G A T C A T G G C T A A T C G C G A T G T A C G C T A A G C T G T A C | Fra1(bZIP)/BT549-Fra1-ChIP-Seq(GSE46166)/Homer | 1e-24 | -5.633e+01 | 0.0000 | 184.0 | 18.40% | 3933.4 | 8.14% | motif file (matrix) | svg |
| 14 | A C T G C A T G T C G A A C G T A C T G C G T A A T C G A C G T G T A C C G T A G A C T A G T C | Fos(bZIP)/TSC-Fos-ChIP-Seq(GSE110950)/Homer | 1e-23 | -5.488e+01 | 0.0000 | 189.0 | 18.90% | 4151.4 | 8.59% | motif file (matrix) | svg |
| 15 | C A T G C T A G T C G A A C G T A C T G C G T A T A G C C G A T T G A C C G T A A G C T G A T C | Fra2(bZIP)/Striatum-Fra2-ChIP-Seq(GSE43429)/Homer | 1e-22 | -5.292e+01 | 0.0000 | 156.0 | 15.60% | 3144.2 | 6.51% | motif file (matrix) | svg |
| 16 | A G T C G A C T C A G T A C T G C T A G T G A C G C T A A T G C G C A T A T C G C G A T A C T G G A T C G T A C G T C A C T G A | NF1(CTF)/LNCAP-NF1-ChIP-Seq(Unpublished)/Homer | 1e-22 | -5.257e+01 | 0.0000 | 101.0 | 10.10% | 1543.8 | 3.20% | motif file (matrix) | svg |
| 17 | C T A G T C G A G C A T C A T G G C T A T A G C C G A T G T A C C T G A A G C T | JunB(bZIP)/DendriticCells-Junb-ChIP-Seq(GSE36099)/Homer | 1e-22 | -5.158e+01 | 0.0000 | 173.0 | 17.30% | 3739.3 | 7.74% | motif file (matrix) | svg |
| 18 | C T A G T C G A C G A T A C T G C G T A T A C G A G C T T G A C G C T A A C G T G A T C T A G C | Fosl2(bZIP)/3T3L1-Fosl2-ChIP-Seq(GSE56872)/Homer | 1e-17 | -4.092e+01 | 0.0000 | 102.0 | 10.20% | 1856.2 | 3.84% | motif file (matrix) | svg |
| 19 | C T A G A G T C T A C G T A C G T G A C C G T A A C T G T A G C G C A T C A T G A T G C A G C T | Ascl1(bHLH)/NeuralTubes-Ascl1-ChIP-Seq(GSE55840)/Homer | 1e-15 | -3.566e+01 | 0.0000 | 253.0 | 25.30% | 7424.5 | 15.37% | motif file (matrix) | svg |
| 20 | C T A G T C G A A C G T A C T G C G T A T A G C C G A T G T A C C G T A A G C T G A T C G T A C | Jun-AP1(bZIP)/K562-cJun-ChIP-Seq(GSE31477)/Homer | 1e-15 | -3.483e+01 | 0.0000 | 78.0 | 7.80% | 1328.2 | 2.75% | motif file (matrix) | svg |
| 21 | C A T G A G T C G A C T C G T A C G A T G C A T G A C T G C A T C G A T C T A G C A T G T G A C | Mef2b(MADS)/HEK293-Mef2b.V5-ChIP-Seq(GSE67450)/Homer | 1e-14 | -3.407e+01 | 0.0000 | 263.0 | 26.30% | 7924.8 | 16.40% | motif file (matrix) | svg |
| 22 | C A T G G T A C G A C T G C T A C G T A C G T A C G T A G C T A G A C T C T G A T C A G G T A C | Mef2c(MADS)/GM12878-Mef2c-ChIP-Seq(GSE32465)/Homer | 1e-11 | -2.693e+01 | 0.0000 | 163.0 | 16.30% | 4490.3 | 9.29% | motif file (matrix) | svg |
| 23 | C A G T T C A G G A T C A C T G A C G T C T A G A C T G A C T G G A C T C T A G | Egr1(Zf)/K562-Egr1-ChIP-Seq(GSE32465)/Homer | 1e-11 | -2.649e+01 | 0.0000 | 88.0 | 8.80% | 1883.4 | 3.90% | motif file (matrix) | svg |
| 24 | A G T C C G A T A C T G A T C G T G A C G C T A C A T G A T C G T G A C C G A T A C T G T A G C G T A C G T C A | Tlx?(NR)/NPC-H3K4me1-ChIP-Seq(GSE16256)/Homer | 1e-11 | -2.636e+01 | 0.0000 | 89.0 | 8.90% | 1921.0 | 3.98% | motif file (matrix) | svg |
| 25 | G T A C G A T C C A G T A G T C A G T C A G T C T G C A G A T C C T G A A T G C G T C A A C G T | WT1(Zf)/Kidney-WT1-ChIP-Seq(GSE90016)/Homer | 1e-10 | -2.384e+01 | 0.0000 | 77.0 | 7.70% | 1628.2 | 3.37% | motif file (matrix) | svg |
| 26 | G T A C G A C T C G T A C T G A T C G A C G T A G C T A C A G T C T G A T A C G | Mef2a(MADS)/HL1-Mef2a.biotin-ChIP-Seq(GSE21529)/Homer | 1e-10 | -2.371e+01 | 0.0000 | 143.0 | 14.30% | 3930.5 | 8.14% | motif file (matrix) | svg |
| 27 | G A C T C T A G A T G C A G T C G T C A T A C G A T G C A T C G | HIC1(Zf)/Treg-ZBTB29-ChIP-Seq(GSE99889)/Homer | 1e-9 | -2.185e+01 | 0.0000 | 286.0 | 28.60% | 9830.1 | 20.35% | motif file (matrix) | svg |
| 28 | A G C T C A T G G C A T G A T C T G C A C T A G G A T C A C G T | Tgif2(Homeobox)/mES-Tgif2-ChIP-Seq(GSE55404)/Homer | 1e-9 | -2.093e+01 | 0.0000 | 568.0 | 56.80% | 22813.4 | 47.22% | motif file (matrix) | svg |
| 29 | A T G C T C G A A G T C A G C T A C G T G T A C A G T C G C T A C T A G C A T G G T C A C T G A T C A G A G T C | Stat3+il21(Stat)/CD4-Stat3-ChIP-Seq(GSE19198)/Homer | 1e-8 | -2.017e+01 | 0.0000 | 150.0 | 15.00% | 4415.7 | 9.14% | motif file (matrix) | svg |
| 30 | T C G A T A G C G T C A A C T G A C T G C G T A C G T A C T A G A G C T T C A G | ERG(ETS)/VCaP-ERG-ChIP-Seq(GSE14097)/Homer | 1e-8 | -1.989e+01 | 0.0000 | 276.0 | 27.60% | 9589.2 | 19.85% | motif file (matrix) | svg |
| 31 | T C A G A G C T A C G T A C G T G T A C G A T C C G T A C T A G C A T G G T C A C G T A T C G A | STAT4(Stat)/CD4-Stat4-ChIP-Seq(GSE22104)/Homer | 1e-8 | -1.924e+01 | 0.0000 | 220.0 | 22.00% | 7291.2 | 15.09% | motif file (matrix) | svg |
| 32 | A G C T A G T C A T G C A G C T A C G T C G T A A C G T A G T C C G A T A T G C | Gata2(Zf)/K562-GATA2-ChIP-Seq(GSE18829)/Homer | 1e-8 | -1.921e+01 | 0.0000 | 180.0 | 18.00% | 5664.4 | 11.73% | motif file (matrix) | svg |
| 33 | T A G C G C T A A C T G C G T A A C G T C G T A C G T A T A C G T C A G T C G A | Gata1(Zf)/K562-GATA1-ChIP-Seq(GSE18829)/Homer | 1e-8 | -1.883e+01 | 0.0000 | 159.0 | 15.90% | 4859.1 | 10.06% | motif file (matrix) | svg |
| 34 | C G A T T G C A T G C A G A T C C G T A A C T G T G A C G A C T C A T G A C T G | Tcf21(bHLH)/ArterySmoothMuscle-Tcf21-ChIP-Seq(GSE61369)/Homer | 1e-8 | -1.842e+01 | 0.0000 | 152.0 | 15.20% | 4614.1 | 9.55% | motif file (matrix) | svg |
| 35 | A T G C G A C T A C T G C A G T G A T C A C G T T A C G T A C G | Smad2(MAD)/ES-SMAD2-ChIP-Seq(GSE29422)/Homer | 1e-7 | -1.835e+01 | 0.0000 | 257.0 | 25.70% | 8928.3 | 18.48% | motif file (matrix) | svg |
| 36 | A G C T A T G C G A C T G C A T C G T A A G C T G T A C C G A T A T C G A G T C | Gata6(Zf)/HUG1N-GATA6-ChIP-Seq(GSE51936)/Homer | 1e-7 | -1.714e+01 | 0.0000 | 242.0 | 24.20% | 8410.9 | 17.41% | motif file (matrix) | svg |
| 37 | C A T G A G T C A G C T C G T A C G A T C G A T G C A T G C A T C G A T C T G A C A T G T G A C | Mef2d(MADS)/Retina-Mef2d-ChIP-Seq(GSE61391)/Homer | 1e-7 | -1.698e+01 | 0.0000 | 73.0 | 7.30% | 1773.9 | 3.67% | motif file (matrix) | svg |
| 38 | G A C T C G A T C T G A G T C A G A C T C G A T T C G A C G T A G C T A G C T A T G A C G T A C C G T A A C T G T G C A C G A T A C T G A C G T | Pitx1:Ebox(Homeobox,bHLH)/Hindlimb-Pitx1-ChIP-Seq(GSE41591)/Homer | 1e-7 | -1.684e+01 | 0.0000 | 74.0 | 7.40% | 1814.7 | 3.76% | motif file (matrix) | svg |
| 39 | G A C T C A G T A G C T C G A T A G T C G A T C A G T C C G T A A T G C T C A G | Rbpj1(?)/Panc1-Rbpj1-ChIP-Seq(GSE47459)/Homer | 1e-6 | -1.609e+01 | 0.0000 | 252.0 | 25.20% | 8945.4 | 18.52% | motif file (matrix) | svg |
| 40 | C T G A A C G T A C G T A C G T A G T C G A C T C G A T C T G A A C T G C G T A C G T A T C G A | STAT5(Stat)/mCD4+-Stat5-ChIP-Seq(GSE12346)/Homer | 1e-6 | -1.584e+01 | 0.0000 | 97.0 | 9.70% | 2690.0 | 5.57% | motif file (matrix) | svg |
| 41 | T C G A A G T C C G T A A T C G T A G C A C G T A C T G A G C T A C G T A G T C | Ptf1a(bHLH)/Panc1-Ptf1a-ChIP-Seq(GSE47459)/Homer | 1e-6 | -1.561e+01 | 0.0000 | 379.0 | 37.90% | 14631.8 | 30.29% | motif file (matrix) | svg |
| 42 | T A C G A T C G T A G C G A T C A C T G A C G T A G T C A C G T C T A G A T C G | Smad4(MAD)/ESC-SMAD4-ChIP-Seq(GSE29422)/Homer | 1e-6 | -1.558e+01 | 0.0000 | 252.0 | 25.20% | 9000.2 | 18.63% | motif file (matrix) | svg |
| 43 | A T G C A G T C C T G A A G T C C G A T A C G T A G T C A G T C A C G T A T C G G A C T A C G T | Etv2(ETS)/ES-ER71-ChIP-Seq(GSE59402)/Homer | 1e-6 | -1.476e+01 | 0.0000 | 163.0 | 16.30% | 5345.2 | 11.06% | motif file (matrix) | svg |
| 44 | G A C T C T A G C T A G A G T C T G C A A C T G A C G T A C G T C T A G T C A G | AMYB(HTH)/Testes-AMYB-ChIP-Seq(GSE44588)/Homer | 1e-6 | -1.426e+01 | 0.0000 | 276.0 | 27.60% | 10194.1 | 21.10% | motif file (matrix) | svg |
| 45 | T C G A T A G C T G C A A C T G A C T G C G T A C G T A C T A G G A C T T A C G | ETS1(ETS)/Jurkat-ETS1-ChIP-Seq(GSE17954)/Homer | 1e-6 | -1.420e+01 | 0.0000 | 177.0 | 17.70% | 5971.7 | 12.36% | motif file (matrix) | svg |
| 46 | C G A T T A C G T G A C G A C T C A T G C G T A T A C G A C G T G T A C C T G A | Bach2(bZIP)/OCILy7-Bach2-ChIP-Seq(GSE44420)/Homer | 1e-6 | -1.391e+01 | 0.0000 | 48.0 | 4.80% | 1069.4 | 2.21% | motif file (matrix) | svg |
| 47 | T A C G A T G C G C T A A C T G C G T A A C G T C G T A C T G A T A C G T C G A | Gata4(Zf)/Heart-Gata4-ChIP-Seq(GSE35151)/Homer | 1e-5 | -1.283e+01 | 0.0000 | 248.0 | 24.80% | 9142.7 | 18.93% | motif file (matrix) | svg |
| 48 | A G T C G C A T C G T A C G T A G T A C A C G T A C T G G A T C G A T C T C G A | BMYB(HTH)/Hela-BMYB-ChIP-Seq(GSE27030)/Homer | 1e-5 | -1.272e+01 | 0.0000 | 275.0 | 27.50% | 10340.5 | 21.40% | motif file (matrix) | svg |
| 49 | A G C T G C A T A C T G A C G T A G T C A C G T C T A G T A C G | Smad3(MAD)/NPC-Smad3-ChIP-Seq(GSE36673)/Homer | 1e-5 | -1.265e+01 | 0.0000 | 457.0 | 45.70% | 18667.4 | 38.64% | motif file (matrix) | svg |
| 50 | G C T A G A C T G A C T T G C A C G T A A G T C C G T A T A G C G A T C G A C T | Eomes(T-box)/H9-Eomes-ChIP-Seq(GSE26097)/Homer | 1e-5 | -1.198e+01 | 0.0001 | 398.0 | 39.80% | 16017.7 | 33.16% | motif file (matrix) | svg |
| 51 | G A C T C A G T G A T C G A T C A C G T G A T C C T G A T A C G C G T A G T C A | STAT6(Stat)/Macrophage-Stat6-ChIP-Seq(GSE38377)/Homer | 1e-5 | -1.173e+01 | 0.0001 | 133.0 | 13.30% | 4396.7 | 9.10% | motif file (matrix) | svg |
| 52 | C A G T T C A G A G C T G A C T A C G T A G T C G A T C G A C T C T G A A C T G G A T C C G T A C T G A A G T C G T A C | Rfx6(HTH)/Min6b1-Rfx6.HA-ChIP-Seq(GSE62844)/Homer | 1e-5 | -1.166e+01 | 0.0001 | 169.0 | 16.90% | 5891.3 | 12.19% | motif file (matrix) | svg |
| 53 | T G A C A T G C C G T A A T C G A T G C C A G T C A T G A C T G A G T C G T A C | HEB(bHLH)/mES-Heb-ChIP-Seq(GSE53233)/Homer | 1e-5 | -1.160e+01 | 0.0001 | 240.0 | 24.00% | 8945.4 | 18.52% | motif file (matrix) | svg |
| 54 | A T G C C T G A G A C T A C G T A C G T G T A C G A T C C G A T C T A G C A T G C G T A C G T A C T G A G A C T | STAT1(Stat)/HelaS3-STAT1-ChIP-Seq(GSE12782)/Homer | 1e-4 | -1.138e+01 | 0.0001 | 81.0 | 8.10% | 2375.0 | 4.92% | motif file (matrix) | svg |
| 55 | C T G A C T G A C A T G A T C G A G C T A T C G G A C T C A T G C T G A G T C A | Tbr1(T-box)/Cortex-Tbr1-ChIP-Seq(GSE71384)/Homer | 1e-4 | -1.134e+01 | 0.0001 | 267.0 | 26.70% | 10169.7 | 21.05% | motif file (matrix) | svg |
| 56 | C T A G A G C T G A C T C A T G A G T C A G T C G T C A C A G T C T A G T C A G G T A C C T G A T C G A G A T C T G A C | Rfx2(HTH)/LoVo-RFX2-ChIP-Seq(GSE49402)/Homer | 1e-4 | -1.113e+01 | 0.0001 | 22.0 | 2.20% | 368.3 | 0.76% | motif file (matrix) | svg |
| 57 | A G T C A T C G C T A G A G C T G A C T C T A G A G T C A G T C G C T A C A G T T C A G T C A G G A T C C T G A T C G A G A T C | RFX(HTH)/K562-RFX3-ChIP-Seq(SRA012198)/Homer | 1e-4 | -1.031e+01 | 0.0003 | 19.0 | 1.90% | 306.2 | 0.63% | motif file (matrix) | svg |
| 58 | C T G A T A G C T G A C T C A G C T A G G T C A C G T A T C A G A G C T T C A G | ETV4(ETS)/HepG2-ETV4-ChIP-Seq(ENCODE)/Homer | 1e-4 | -1.003e+01 | 0.0003 | 146.0 | 14.60% | 5102.8 | 10.56% | motif file (matrix) | svg |
| 59 | G T C A T G C A G C T A A G T C C G T A A C T G T G A C G C A T T C A G C A G T | Ap4(bHLH)/AML-Tfap4-ChIP-Seq(GSE45738)/Homer | 1e-4 | -9.844e+00 | 0.0004 | 176.0 | 17.60% | 6393.8 | 13.23% | motif file (matrix) | svg |
| 60 | A C T G T C A G A G C T G A C T C A T G A G T C A G T C G C T A C G A T C T A G T C A G G T A C C T G A T C G A | Rfx1(HTH)/NPC-H3K4me1-ChIP-Seq(GSE16256)/Homer | 1e-4 | -9.831e+00 | 0.0004 | 39.0 | 3.90% | 942.5 | 1.95% | motif file (matrix) | svg |
| 61 | T C A G G A C T C A G T C T G A A G C T C T A G G A C T T G C A C T G A A G T C | HLF(bZIP)/HSC-HLF.Flag-ChIP-Seq(GSE69817)/Homer | 1e-4 | -9.763e+00 | 0.0004 | 215.0 | 21.50% | 8092.7 | 16.75% | motif file (matrix) | svg |
| 62 | A G T C G A C T C A G T G T A C A G T C A T C G T C A G A C T G G T C A C G T A | Stat3(Stat)/mES-Stat3-ChIP-Seq(GSE11431)/Homer | 1e-4 | -9.514e+00 | 0.0005 | 89.0 | 8.90% | 2825.7 | 5.85% | motif file (matrix) | svg |
| 63 | T G C A C T G A A T G C G T C A A C T G A C T G C G T A C G T A C T A G A G C T | Ets1-distal(ETS)/CD4+-PolII-ChIP-Seq(Barski\_et\_al.)/Homer | 1e-3 | -9.143e+00 | 0.0007 | 61.0 | 6.10% | 1771.9 | 3.67% | motif file (matrix) | svg |
| 64 | C G T A C G T A C G T A G C A T G C A T T A C G G T A C G A C T A C T G C G T A A T C G A C G T G T A C C G T A A G C T | Bach1(bZIP)/K562-Bach1-ChIP-Seq(GSE31477)/Homer | 1e-3 | -8.737e+00 | 0.0011 | 20.0 | 2.00% | 375.6 | 0.78% | motif file (matrix) | svg |
| 65 | C T G A T C A G A G T C C G T A A T C G A T G C C G A T A C T G A G T C G A C T A T C G A G T C | MyoD(bHLH)/Myotube-MyoD-ChIP-Seq(GSE21614)/Homer | 1e-3 | -8.728e+00 | 0.0011 | 94.0 | 9.40% | 3093.0 | 6.40% | motif file (matrix) | svg |
| 66 | T C G A C T G A T A G C T G A C T C A G T C A G C G T A C G T A T C A G A G C T | ETV1(ETS)/GIST48-ETV1-ChIP-Seq(GSE22441)/Homer | 1e-3 | -8.648e+00 | 0.0012 | 199.0 | 19.90% | 7545.9 | 15.62% | motif file (matrix) | svg |
| 67 | C G T A A C T G C G T A A C G T C G T A C T G A T C A G T G C A A G C T T G A C | TRPS1(Zf)/MCF7-TRPS1-ChIP-Seq(GSE107013)/Homer | 1e-3 | -8.280e+00 | 0.0017 | 468.0 | 46.80% | 19956.2 | 41.31% | motif file (matrix) | svg |
| 68 | T C G A T C G A T A G C G T A C T C A G T A C G C G T A C G T A T C A G A G C T | GABPA(ETS)/Jurkat-GABPa-ChIP-Seq(GSE17954)/Homer | 1e-3 | -8.222e+00 | 0.0017 | 127.0 | 12.70% | 4505.3 | 9.33% | motif file (matrix) | svg |
| 69 | C T G A A G C T A C G T A C G T A G T C G A C T G A C T C T G A C T G A C T A G C G T A C G T A | STAT6(Stat)/CD4-Stat6-ChIP-Seq(GSE22104)/Homer | 1e-3 | -8.143e+00 | 0.0019 | 127.0 | 12.70% | 4514.7 | 9.35% | motif file (matrix) | svg |
| 70 | T C G A A C T G A C T G C G T A C G T A T C G A A G T C C T G A A T C G G T A C G C A T C A T G | ETS:E-box(ETS,bHLH)/HPC7-Scl-ChIP-Seq(GSE22178)/Homer | 1e-3 | -8.123e+00 | 0.0019 | 21.0 | 2.10% | 424.4 | 0.88% | motif file (matrix) | svg |
| 71 | A G T C C T G A A G T C C G A T C A G T G A T C A T G C A C T G A T C G G A C T | Fli1(ETS)/CD8-FLI-ChIP-Seq(GSE20898)/Homer | 1e-3 | -8.006e+00 | 0.0021 | 155.0 | 15.50% | 5722.7 | 11.85% | motif file (matrix) | svg |
| 72 | G C T A A T C G G C T A G A C T G C T A T C G A T A G C T C G A | GATA3(Zf)/iTreg-Gata3-ChIP-Seq(GSE20898)/Homer | 1e-3 | -7.965e+00 | 0.0021 | 354.0 | 35.40% | 14664.5 | 30.36% | motif file (matrix) | svg |
| 73 | C G T A T A G C T A G C T G C A A C T G C T A G C G T A C G T A T C A G G A C T | EHF(ETS)/LoVo-EHF-ChIP-Seq(GSE49402)/Homer | 1e-3 | -7.964e+00 | 0.0021 | 221.0 | 22.10% | 8617.1 | 17.84% | motif file (matrix) | svg |
| 74 | T C G A A C G T A C G T C T G A G A T C T C A G G A C T G T C A C G T A A G C T G T C A C T A G A G C T A C G T T C G A | NFIL3(bZIP)/HepG2-NFIL3-ChIP-Seq(Encode)/Homer | 1e-3 | -7.656e+00 | 0.0028 | 155.0 | 15.50% | 5768.5 | 11.94% | motif file (matrix) | svg |
| 75 | A C G T T A C G G A T C A C T G A C G T C T A G A C T G A C T G G A T C C T A G C A T G C T A G | Egr2(Zf)/Thymocytes-Egr2-ChIP-Seq(GSE34254)/Homer | 1e-3 | -7.626e+00 | 0.0029 | 19.0 | 1.90% | 379.3 | 0.79% | motif file (matrix) | svg |
| 76 | C A T G C T A G A G C T G A C T C A T G A G T C G A T C G C T A C G A T C T A G T C A G G T A C C T G A T C G A | X-box(HTH)/NPC-H3K4me1-ChIP-Seq(GSE16256)/Homer | 1e-3 | -7.473e+00 | 0.0033 | 27.0 | 2.70% | 642.0 | 1.33% | motif file (matrix) | svg |
| 77 | G A C T G T A C T G C A A C G T G A T C G C T A T C G A A C G T A G T C C G T A | Pdx1(Homeobox)/Islet-Pdx1-ChIP-Seq(SRA008281)/Homer | 1e-3 | -7.460e+00 | 0.0033 | 243.0 | 24.30% | 9683.0 | 20.04% | motif file (matrix) | svg |
| 78 | C T A G C T A G A G T C T C A G A C T G A C G T A C G T C T G A | MYB(HTH)/ERMYB-Myb-ChIPSeq(GSE22095)/Homer | 1e-3 | -7.284e+00 | 0.0039 | 295.0 | 29.50% | 12075.7 | 25.00% | motif file (matrix) | svg |
| 79 | T G C A A G C T A C G T C T A G G A T C C T A G G A T C G T C A C T G A A G T C | CEBP(bZIP)/ThioMac-CEBPb-ChIP-Seq(GSE21512)/Homer | 1e-3 | -7.268e+00 | 0.0039 | 147.0 | 14.70% | 5475.2 | 11.33% | motif file (matrix) | svg |
| 80 | C A T G A C T G C T A G T C G A T C G A T C G A T C G A T C A G T C A G T C A G T G A C T G A C C G T A A C T G T G C A C G A T A C T G | RBPJ:Ebox(?,bHLH)/Panc1-Rbpj1-ChIP-Seq(GSE47459)/Homer | 1e-3 | -6.996e+00 | 0.0050 | 58.0 | 5.80% | 1812.8 | 3.75% | motif file (matrix) | svg |
| 81 | G A C T C G A T T C A G G A T C G A C T A G C T A G C T A G T C G A T C C G T A C T A G C T A G T C G A T C G A C T G A | Bcl6(Zf)/Liver-Bcl6-ChIP-Seq(GSE31578)/Homer | 1e-3 | -6.960e+00 | 0.0052 | 259.0 | 25.90% | 10494.1 | 21.72% | motif file (matrix) | svg |
| 82 | C G T A C T G A C G T A C T A G T C G A C T A G A C T G C G T A C G T A T A C G A G C T A T C G | SpiB(ETS)/OCILY3-SPIB-ChIP-Seq(GSE56857)/Homer | 1e-2 | -6.826e+00 | 0.0058 | 49.0 | 4.90% | 1477.5 | 3.06% | motif file (matrix) | svg |
| 83 | A C G T T C G A T C G A A G T C G T C A T A C G A T G C A C G T A C T G A G C T | Myf5(bHLH)/GM-Myf5-ChIP-Seq(GSE24852)/Homer | 1e-2 | -6.598e+00 | 0.0072 | 91.0 | 9.10% | 3183.6 | 6.59% | motif file (matrix) | svg |
| 84 | C T G A C T A G A C T G G C A T C T A G G C A T A T C G C T G A C G T A G T C A | Tbx21(T-box)/GM12878-TBX21-ChIP-Seq(Encode)/Homer | 1e-2 | -6.478e+00 | 0.0081 | 175.0 | 17.50% | 6813.9 | 14.10% | motif file (matrix) | svg |
| 85 | C T G A C T A G A T C G G C A T A C T G G T A C A T G C C G T A A C T G G C T A A G T C C G T A | Tbox:Smad(T-box,MAD)/ESCd5-Smad2\_3-ChIP-Seq(GSE29422)/Homer | 1e-2 | -6.338e+00 | 0.0092 | 39.0 | 3.90% | 1132.0 | 2.34% | motif file (matrix) | svg |
| 86 | T C A G T A C G T A G C A C G T A C T G C G A T A G T C C G T A T A C G A G T C | Meis1(Homeobox)/MastCells-Meis1-ChIP-Seq(GSE48085)/Homer | 1e-2 | -6.289e+00 | 0.0095 | 253.0 | 25.30% | 10347.4 | 21.42% | motif file (matrix) | svg |
| 87 | C T G A C G A T C T A G C G T A A G C T C G A T C A G T C T G A G A C T C T A G C T A G A T G C | PBX2(Homeobox)/K562-PBX2-ChIP-Seq(Encode)/Homer | 1e-2 | -6.235e+00 | 0.0099 | 209.0 | 20.90% | 8368.3 | 17.32% | motif file (matrix) | svg |
| 88 | T G C A C T G A A G T C G T C A A C T G A C T G C G T A C G T A C T G A A G C T | EWS:FLI1-fusion(ETS)/SK\_N\_MC-EWS:FLI1-ChIP-Seq(SRA014231)/Homer | 1e-2 | -6.006e+00 | 0.0123 | 92.0 | 9.20% | 3292.2 | 6.81% | motif file (matrix) | svg |
| 89 | C T G A A G T C C G A T A G C T A T G C G T A C A C G T A T C G C A G T G C A T | Elf4(ETS)/BMDM-Elf4-ChIP-Seq(GSE88699)/Homer | 1e-2 | -5.842e+00 | 0.0144 | 146.0 | 14.60% | 5637.6 | 11.67% | motif file (matrix) | svg |
| 90 | C T A G T C A G C A G T T C A G A C T G A C T G G A T C C T A G A C T G C T A G T C A G A T G C | KLF14(Zf)/HEK293-KLF14.GFP-ChIP-Seq(GSE58341)/Homer | 1e-2 | -5.452e+00 | 0.0210 | 125.0 | 12.50% | 4779.1 | 9.89% | motif file (matrix) | svg |
| 91 | T G A C G T A C C G T A A C T G T G A C C G A T A C T G A T C G A G C T T A C G T C G A T A G C G T A C C G T A A T C G T G A C G C A T A C T G A C T G A T G C | Twist(bHLH)/HMLE-TWIST1-ChIP-Seq(Chang\_et\_al)/Homer | 1e-2 | -5.423e+00 | 0.0213 | 22.0 | 2.20% | 566.1 | 1.17% | motif file (matrix) | svg |
| 92 | T C G A A G C T A C G T A C G T A G T C A G T C A C G T A T C G G A C T A T C G | EWS:ERG-fusion(ETS)/CADO\_ES1-EWS:ERG-ChIP-Seq(SRA014231)/Homer | 1e-2 | -5.289e+00 | 0.0241 | 140.0 | 14.00% | 5461.9 | 11.31% | motif file (matrix) | svg |
| 93 | C G T A C T A G C A T G A G C T A C T G C G A T A T C G C G T A G T C A G T C A | Tbet(T-box)/CD8-Tbet-ChIP-Seq(GSE33802)/Homer | 1e-2 | -4.866e+00 | 0.0364 | 188.0 | 18.80% | 7679.7 | 15.90% | motif file (matrix) | svg |
| 94 | T A C G T C G A C A G T A C T G G C T A A T G C C G A T G T A C C G T A A C T G T A G C C G T A | NF-E2(bZIP)/K562-NFE2-ChIP-Seq(GSE31477)/Homer | 1e-2 | -4.825e+00 | 0.0376 | 15.0 | 1.50% | 353.7 | 0.73% | motif file (matrix) | svg |
| 95 | T C G A T C G A A G T C C G T A C T A G T A G C A C G T A C T G | MyoG(bHLH)/C2C12-MyoG-ChIP-Seq(GSE36024)/Homer | 1e-2 | -4.763e+00 | 0.0396 | 123.0 | 12.30% | 4799.8 | 9.94% | motif file (matrix) | svg |
| 96 | G A C T G C A T C T A G C G A T G A T C T C G A C A T G G A T C | Tgif1(Homeobox)/mES-Tgif1-ChIP-Seq(GSE55404)/Homer | 1e-2 | -4.748e+00 | 0.0397 | 500.0 | 50.00% | 22317.0 | 46.20% | motif file (matrix) | svg |
| 97 | T C G A A G T C C G T A A T C G A T G C C G A T A C T G A G T C A G C T A C T G | Tcf12(bHLH)/GM12878-Tcf12-ChIP-Seq(GSE32465)/Homer | 1e-2 | -4.683e+00 | 0.0420 | 101.0 | 10.10% | 3851.2 | 7.97% | motif file (matrix) | svg |
| 98 | C T G A A T G C C G T A A C G T A G T C A G T C A C G T A C T G A T C G G C A T | SPDEF(ETS)/VCaP-SPDEF-ChIP-Seq(SRA014231)/Homer | 1e-2 | -4.679e+00 | 0.0420 | 160.0 | 16.00% | 6456.6 | 13.37% | motif file (matrix) | svg |
